# Minimal instructions improve the performance of laypersons in the use of semiautomatic and automatic external defibrillators

**DOI:** 10.1186/cc3033

**Published:** 2005-01-31

**Authors:** Stefan Beckers, Michael Fries, Johannes Bickenbach, Matthias Derwall, Ralf Kuhlen, Rolf Rossaint

**Affiliations:** 1 Resident, Department of Anaesthesiology, University Hospital Aachen, Aachen, Germany; 2 Medical Student, Department of Anaesthesiology, University Hospital Aachen, Aachen, Germany; 3 Professor, Department of Anaesthesiology, University Hospital Aachen, Aachen, Germany; 4 Professor and Chairman, Department of Anaesthesiology, University Hospital Aachen, Aachen, Germany

**Keywords:** automated external defibrillator, cardiopulmonary resuscitation, defibrillation, layperson, intuitive

## Abstract

**Introduction:**

There is evidence that use of automated external defibrillators (AEDs) by laypersons improves rates of survival from cardiac arrest, but there is no consensus on the optimal content and duration of training for this purpose. In this study we examined the use of semiautomatic or automatic AEDs by laypersons who had received no training (intuitive use) and the effects of minimal general theoretical instructions on their performance.

**Methods:**

In a mock cardiac arrest scenario, 236 first year medical students who had not previously attended any preclinical courses were evaluated in their first study week, before and after receiving prespecified instructions (15 min) once. The primary end-point was the time to first shock for each time point; secondary end-points were correct electrode pad positioning, safety of the procedure and the subjective feelings of the students.

**Results:**

The mean time to shock for both AED types was 81.2 ± 19.2 s (range 45–178 s). Correct pad placement was observed in 85.6% and adequate safety in 94.1%. The time to shock after instruction decreased significantly to 56.8 ± 9.9 s (range 35–95 s; P ≤ 0.01), with correct electrode placement in 92.8% and adequate safety in 97%. The students were significantly quicker at both evaluations using the semiautomatic device than with the automatic AED (first evaluation: 77.5 ± 20.5 s versus 85.2 ± 17 s, P ≤ 0.01; second evaluation: 55 ± 10.3 s versus 59.6 ± 9.6 s, P ≤ 0.01).

**Conclusion:**

Untrained laypersons can use semiautomatic and automatic AEDs sufficiently quickly and without instruction. After one use and minimal instructions, improvements in practical performance were significant. All tested laypersons were able to deliver the first shock in under 1 min.

## Introduction

Mortality from sudden cardiac death is up to 375,000 patients per year in Europe [1] and in the vast majority of cases it is caused by ventricular fibrillation [2]. To increase survival rates, the period between developing ventricular fibrillation and the first defibrillation must be as short as possible. Early defibrillation, done during the first minute of the event, is successful in 85% of cases. Each additional minute without treatment reduces the survival rate by a further 10% [3]. Therefore, early defibrillation must be implemented into the chain of survival [4], and to this end the development of programmes for nonmedical lay responders is recommended and supported by many international societies. For years, the American Heart Association has postulated inclusion of AED use in basic life support (BLS) training [4,5]. Furthermore, first responders may operate an AED without having any background knowledge about the instrument. Previous studies have shown that even children can handle an AED confidently and effectively [6].

There is no consensus as yet regarding time frames for specific training programmes, but for organizational reasons and for further implementation of public access defibrillation (PAD) programmes in the future, it is necessary that this period be defined. It remains unclear how lay users should be instructed to perform safe and effective defibrillation. The aim of the present study was to evaluate the intuitive use (i.e. without training) of AEDs, both in fully automatic and in semiautomatic modes, and to study the effect of brief and well directed theoretical instruction.

## Methods

### Participants

A total of 236 first year medical students were tested during their first 2 weeks at the medical school of the University of Aachen. All students were informed that their performance would be evaluated and used for scientific study. No personal data were collected. Furthermore, no damage to anyone's health was anticipated because the AED uses no current. Therefore, the institutional ethical committee waived the need to obtain informed consent from each participant. None of the students were prompted or prepared in any way before the study.

### Equipment

The Medtronic Physio-Control LifePak™ CR-T AED trainer (Medtronic Physio-Control LifePak™; Medtronic, Düsseldorf, Germany) provides the necessary interface for demonstrating practical skills during a simulated cardiac arrest, and was used rather than the original Medtronic Physio-Control LifePak™ CR Plus. No current is applied by the training device.

**Figure 1:**
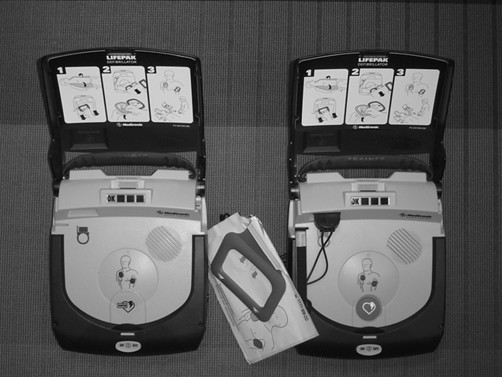
Evaluated automated external defibrillator: (left) automatic mode and (right) semiautomatic mode. Weight: 2.1 kg; physical dimensions: 10 × 20 × 24 cm.

After opening the lid a red handle must be pulled, which then releases self-adhesive electrode pads with integrated cables connected to the device (Fig. 1). Voice prompts (Table 1) and an illustrated reference card inside the opened lid guides users in a step-by-step manner. No text prompts are displayed on the screen. After turning the device on and positioning the electrodes properly, the analyzing process of the AED starts automatically and is finished after 10 s in both types of AED. In the semiautomatic mode it takes 18 s from the beginning of the analyzing process until the device is charged, and an alarm tone sounds until the shock button is pressed. In the automatic mode the shock is delivered automatically after 21 s and the charge is calculated from the analysis of heart rhythm over this period [7].

### Study protocol

In a mock cardiac arrest scenario, the students were evaluated on a manikin (ResusciAnne^®^; Laerdal, Stavanger, Norway). After randomization, 118 students were tested on an AED in automatic mode, and 118 were tested on a semiautomatic AED. The device was kept in its usual standby mode. The manikin was positioned supine and dressed in a zippered jacket. Three physicians skilled in providing and teaching advanced life support (certified instructors of the European Resuscitation Council) were present and recorded data while each student operated the AED. Each student was tested individually and was unable to observe the performance of other participants. They were read the following text: 'This patient is unconscious, not breathing and has no signs of circulation. The device in front of you may help to restore spontaneous circulation.'

The procedure ended when the first shock was delivered or no shock could be given in 240 s. Placement of the electrode pads was accepted as correct if the left pad covered at least 50% of an area circumscribed by the nipple line superiorly, costal margin inferiorly, mid-clavicular line medially and mid-axillary line laterally. The right pad was required to cover at least 50% of an area circumscribed by the clavicle superiorly, nipple line inferiorly, anterior axillary line laterally and right sternal margin medially. Application of the AED was considered to be safe if the student remained clear of the manikin during delivery of the shock. If a technical problem occurred, the student damaged the AED, started with conventional cardiopulmonary resuscitation, or had language difficulties, then this was classified as 'any other problem'.

After having completed the tests, each student completed a standardized questionnaire to evaluate whether they had any experience with an AED before the study or whether they had any medical education (e.g. nurse, paramedic etc.). After a period of 1 week all test candidates were assigned the same type of device they had used in their first test and were re-evaluated in the same scenario. During this week they attended a short lecture (15 min) emphasizing the following core objectives: importance of sudden cardiac death and of defibrillation in this context; importance of 'time to shock' to return of spontaneous circulation and success of resuscitation over time; importance of correct electrode pad positioning; safety aspects when using an AED; general procedure for defibrillation devices (e.g. analysis, voice prompts); general AED algorithm following guidelines; and special instructions for slim and overweight victims.

There were no practical training sessions available between the two evaluations and no specific information on the tested AED devices was given.

### Data analysis

Data are expressed as means ± standard deviation. P ≤ 0.05 was considered statistically significant. Statistical software SPSS version 11.0 (SPSS Inc., Chicago, IL, USA) was used.

#### Primary end-points

The primary end-point was to determine the time from the beginning of the scenario to first shock. Using a t-test, differences in time to shock between the first and second evaluations were calculated, as well as between the semiautomatic and the automatic devices for each time point.

#### Secondary end-points

The secondary end-points were chosen to assess correct electrode pad positioning and the safety of the procedure, as well as previous medical knowledge. Data were compared in a proportional manner and tested for significant differences using the McNemar test.

## Results

The mean age of the study population was 20.7 ± 2.9 years (range 18–42 years). Of the 236 students included, 28 (11.9%) had a history of medical education (16 emergency medicine technicians and paramedics, and 12 nurses).

### Time to defibrillation, electrode pad positioning and safety

In the first evaluation the time to shock for both devices was 81.2 ± 19.2 s (range 45–178 s). The pads were positioned correctly by 85.6% of the students. Shock was administered safely by 94.1%. In the second evaluation the time to first defibrillation decreased significantly to 56.8 ± 9.9 s (range 35–95 s; *P *≤ 0.01). The electrodes were correctly placed in 92.8% of cases, and shock was administered safely in 97% of cases.

Table 2 summarizes these variables by type of AED. When comparing time to first shock between semiautomatic and automatic AEDs, the students were significantly faster in both evaluations using the semiautomatic device (first evaluation: 77.5 ± 20.5 s versus 85.2 ± 17 s, *P *≤ 0.01; second evaluation: 55 ± 10.3 s versus 59.6 ± 9.6 s, *P *≤ 0.01).

In the second evaluation 113 out of 118 (95.8%) students were able to deliver a shock safely and none failed in the semiautomatic group. In the automatic group 115 of 118 (97.5%) were able to deliver a shock, but three students failed.

Students with pre-existing medical education were significant faster at both times (first evaluation: 73.0 ± 17.1 s versus 83.0 ± 19.1 s, *P *≤ 0.01; second evaluation: 51.8 ± 9.2 s versus 58.3 ± 10.1 s, *P *≤ 0.01). All other findings are summarized in Table 2.

## Discussion

This study represents the first comparison in laypersons of the use of fully automatic devices with that of semiautomatic devices, including the largest study group yet reported. The improvements with both devices, in terms of time to first shock, between initial use without instruction and use following the described 15-min theoretical instruction were significant.

Since the first clinical use of AEDs in the early 1980s [8], developments in technology have led to initiatives by health and governmental organizations to develop PAD programmes [9]. Various studies [10-13] have shown improvements in rates of survival from out-of-hospital cardiac arrest where nonmedical personnel were trained in PAD programmes. However, only a few studies described the performance of laypersons, but even these individuals were initially instructed before evaluation [6,14]. In a cross-over design, Eames and coworkers [15] compared the use of three different AEDs by nearly untrained laypersons (*n *= 24), but information had been given concerning the application of a shock, following instructions for the device and the impact that time to defibrillation has on outcome. To our knowledge, the present study is the first to describe the use of AEDs without any instructions before first use. It is noteworthy that, even without instruction, 226 out of 236 participants (95.8%) were able to deliver a shock.

Safety aspects associated with automatic mode have been considered and critically discussed, but the question of whether it is better not to administer an advised shock in the case of proven ventricular fibrillation or to have a shock delivered automatically with a delay is rhetorical. Surveying safety aspects of the tested AED, we found that 92.4% of students were able to deliver a shock safely in semiautomatic mode and 95.8% in automatic mode during testing without prior instruction. After theoretical instruction, these rates increased to 95.8% and 97.5%, respectively. Eames and coworkers [15] found that all individuals stood clear while delivering the shock but, as mentioned above, only 24 subjects were tested; it follows that possible reluctance to adhere to safety procedures might not have been detected in that investigation. Fromm and Varon [16] found that still 10 months after initial training, the 'simplicity of use of the particular AED' was the core issue determining safety. The important benefit of devices programmed in automatic mode is that they relieve the layperson of decision making in an unfamiliar and stressful situation.

Contrary to expectations were our findings regarding electrode pad placement. There was an anticipated and significant improvement in the automatic group, but only a trend was observed in the semiautomatic group. It is inexplicable why, after instruction, 9.3% (11 students) still could not achieve correct pad positioning. This is in contrast to the study by Gundry and coworkers [6], in which all children were able to position the pad in the required area, whereas Eames and coworkers [15] observed 20.9% incorrect electrode placement with the LifePak CR Plus. With the Philips/Laerdal Heartstart1 the result was only 4.2%, and the Zoll AEDPlus had the worst result, with 41.6% incorrect pad positioning. In some cases, confusing descriptions or drawings might have caused the incorrect positioning of the adhesive pad electrodes in the present study. Overall, this supports the conclusion of Eames and coworkers that simple devices should be developed with clear visual instructions, and it reiterates that design, construction and visual aids have an impact on user performance. This statement was confirmed by our observation that even in the second evaluation, in the automatic group three students were unable to deliver a shock. In these three cases the students were confused by the voice prompts of the automatic device, and while trying to push the shock button they turned the device off. Other detected problems in both testing sessions occurred mainly as a result of language problems, but they were reduced after instruction. In general, none of the participants appeared to be apprehensive about operating the AED because none of them refused to participate in the study or to apply the device to the manikin.

The significant difference in time to shock before and after instruction between semiautomatic AED and the automatic device is a possible effect of the software version used. However, the programmed delay of 3 s to delivery of shock in the automatic device does not adequately explain this finding. Changing the timing of voice prompts and development of clearer instructions may lead to different results. In general, however, the voice prompts that lead to the best results remains a matter for discussion.

The studies published thus far led to the statement from the American Heart Association and the Resuscitation Council UK 'not to specify the nature of content or duration of BLS plus AED programs due to the lack of current evidence on which to base any such guidance' [17]. As yet there is no consensus regarding the optimal duration of specific training programmes. It will be difficult to achieve that perfect performance of certain skills that indicates successful training of laypersons. Especially for organizational reasons, it is fundamental to define time frames of course concepts. We endorse the assertion by Gundry and coworkers [6] and Moule and Albarran [17] that simplified training programmes should be developed, exploiting the potential of multimedia technology, along with adequate teaching and learning materials.

Various concepts have been described [10,11,17-21], but no data exist regarding how best to train and what the optimal duration of training is to achieve the best outcomes. Moule and Albarran [17] recently stated that the duration and most effective methods for teaching professionals and laypersons remain undefined. For this reason, no recommendation can yet be given. The implementation of PAD programmes in the future will depend mainly on the willingness of the public to participate in AED or cardiopulmonary resuscitation courses. The more time required to achieve the necessary skills, the less people will feel able to participate voluntarily. Furthermore, training sessions must be as precise and short as possible for organizational reasons; ideally, it should be possible for even a small number of instructors to reach a large group of trainees in minimal time.

### Limitations of the study

The groups evaluated here are not representative of the general population with respect to sex (male 35% [*n *= 83], female 65% [*n *= 153]) and age, but the two groups are comparable (Table 2). In addition, the students were not chosen by random; nevertheless, they do represent young and inexperienced laypersons with respect to medical issues because, in Germany, students begin medical school directly after graduation from secondary school, without any specific preparation.

As considered by other studies [16,22], the participants might not have been free from external or internal motivations because of the fact that they were going into medicine. However, at this stage they are at best minimally trained and are not representative of the health care professional community. Furthermore, this internal motivation could have influenced their knowledge of theoretical issues concerning defibrillation within the evaluation period, but it is unlikely that there would have been a significant improvement in practical performance after, for instance, a web search.

Finally, no manikin used to represent an unconscious, breathless and pulseless victim can simulate a human perfectly. Because of this limitation, it is debatable whether benefits obtained in a simulated representation of a complex situation can be realized in clinical practice.

## Conclusions

Untrained laypersons are able to use AEDs quickly and safely. The observation that measures of practical performance (i.e. time to first shock, accuracy of electrode pad placement and safety) were significantly improved after minimal theoretical instruction and one use, but without technical instructions in the use of the specific device, is supportive of widespread implementation of PAD programmes wherever possible. Moreover, enhanced acceptance of AEDs and the increased likelihood that AEDs will be used following directed 'public information' (e.g. television campaigns or other extensive publicly available media) is of great importance. Core issues (e.g. the significance of sudden cardiac death and the importance of defibrillation in this context) should be at the forefront of new educational changes; some suggestions in this regard were made in the present study.

Taken together, our findings support previous recommendations to develop features that can be made available in all AEDs. Sophisticated devices with simple instructions – visual or vocal – should be implemented in PAD programmes. Further developments should aim at simplifying the application of electrodes and achieving consistency in the instructions given by the different manufacturers. Value must be attached to giving general instructions and information about features that are common to all devices; describing the specific details of a device does not appear to be essential, as was assumed.

In our opinion, one of the most remarkable findings is that all tested laypersons were able to deliver a shock in less than 1 min after minimal instructions had been given, regardless of whether automatic or semiautomatic mode was used. Despite the limitations of the study, we conclude that only minimal background knowledge is needed for laypersons to use an AED safely and quickly, and that further implementation of AEDs for use by minimally trained persons without any medical training is possible. We believe that keeping instructions for laypersons as simple as possible will lead to greater acceptance and motivation, and will further facilitate PAD programmes. Time spent training to acquire necessary cardiopulmonary resuscitation skills within the BLS algorithm can be saved by focusing AED instructions in this way. Further studies are warranted to determine whether skills are retained over the long term.

## Key messages

• 	This first observation in 'fully' automatic devices confirms that this type of AED can be used safely and effectively by lay responders.

• 	All tested laypersons were able to deliver a shock in less than 1 min after minimal instructions, regardless of whether automatic or semiautomatic mode was used.

• 	In future value must be attached to general instruction and similarities; describing specific details of available devices is not essential.

• 	Previous recommendations to develop features that can be made available in all AEDs are supported by our findings.

## Abbreviations

AED = automated external defibrillator; BLS = basic life support; PAD = public access defibrillation.

## Competing interests

The author(s) declare that they have no competing interests.

## Authors' contributions

SB had conceived the study. SB, MF, JB, RK and RR designed the study protocol. Testing was performed by SB, MF, JB and MD. Statistical analysis was done by MF and MD. SB, MF, JB, RK and RR wrote and reviewed the manuscript before submission. All authors read and approved the final manuscript.

## Figures and Tables

**Table 1:** Voice prompts of the automated external defibrillator during the simulated cardiac arrest scenario

Automatic	Semiautomatic	Tones
Call for help now		After the AED lid opens, two beeping tones sound. The voice prompts will sound following the beeping tones
Remove clothing from chest		
Pull red handle to open bag		
Peel each pad off blue plastic		
Apply pads to exposed chest		
Do not touch patient – evaluating heart rhythm		Two beeping tones sound to simulate heart rhythm analysis
Stand by – preparing to shock		
Everyone clear		
	Press flashing button	Semiautomatic model only; an alarm tone sounds until the shock button is pressed
Do not touch patient – delivering shock		Automatic model only; an alarm tone sounds until shock is delivered automatically
Shock delivered		
		
**Voice prompts that are not used**		
No shock advised		
Shock not delivered		
Check for pulse; if no pulse start CPR		
Check for breathing; if not breathing start CPR		
Check for signs of circulation; if no signs of circulation start CPR		
Continue care		
Check pads for good contact		
Motion detected		
Stop motion		

AED, automated external defibrillator; CPR, cardiopulmonary resuscitation. Data from Medtronic [7].

**Table 2:** Time to first shock, correct electrode pad positioning and safety aspects before and after brief general instruction in defibrillation

	Device	
	
	Semiautomatic	Automatic
*n*	118	118
Age (mean ± SD)	21.1 ± 3.3	20.4 ± 2.3
Male (*n *[%])	45 (38.1%)	38 (32.2%)
First evaluation		
Time to shock (s; mean ± SD)	77 ± 20.4*^†^	85 ± 17.2^†^
Not able to deliver shock (*n *[%])	6 (5.1%)^‡^	4 (3.4%)
Incorrect pad positioning (*n *[%])	15 (12.7%)	19 (16.1%)^‡^
Safe shock (*n *[%])	109 (92.4%)	113 (95.8%)
Any other problems^a ^(*n *[%])	18 (15.3%)	12 (10.2%)
Second evaluation		
Time to shock (s; mean ± SD)	55 ± 10.3*	59 ± 9.1
Not able to deliver shock (*n *[%])	0 (0%)	3 (2.5%)
Incorrect pad positioning (*n *[%])	11 (9.3%)	3 (2.5%)
Safe shock (*n *[%])	113 (95.8%)	115 (97.5%)
Any other problems^a ^(*n *[%])	3 (2.5%)	2 (1.7%)

Comparison of subjects using semiautomatic and automatic devices at different evaluations. ^a^Any other problems as described in the study protocol. *P < 0.05, versus automatic device (t-test). ^†^*P *< 0.05, versus second evaluation (t-test). ^‡^*P *< 0.05, versus second evaluation (McNemar test). SD, standard deviation.
